# Clinical trajectory and determinants of pulmonary hemorrhage in neonates: a 10-year case–control study

**DOI:** 10.1186/s12887-026-07190-x

**Published:** 2026-07-18

**Authors:** Ayna Atayeva, Ozge Serce Pehlevan

**Affiliations:** 1 Division of Pediatrics,Faculty of Medicine, Kocaeli University, Kocaeli, Türkiye; 2 Department of Neonatology, Division of Pediatrics, Faculty of Medicine, Kocaeli University School of Medicine, Kocaeli, Türkiye

**Keywords:** Cardiorespiratory instability, Fluid imbalance, Neonate, Patent ductus arteriosus, Pulmonary hemorrhage

## Abstract

**Background:**

Pulmonary hemorrhage remains a life-threatening condition in neonates. This study aimed to identify factors associated with pulmonary hemorrhage, characterize the clinical trajectory during the 24 h preceding the event, and evaluate factors associated with mortality.

**Methods:**

In this 10-year single-center case–control study with frequency-matched control selection, neonates with pulmonary hemorrhage were compared with controls matched at the sampling stage according to gestational age, birth weight, and admission period. Perinatal and postnatal variables were analyzed together with clinical characteristics observed during the 24 h preceding pulmonary hemorrhage. Multivariate logistic regression was used to evaluate associations between clinical variables, pulmonary hemorrhage, and mortality. A sensitivity analysis restricted to a recent cohort was performed to assess the robustness of the findings.

**Results:**

A total of 120 neonates (60 cases, 60 controls) were included. Antenatal corticosteroid exposure was associated with a lower likelihood of pulmonary hemorrhage (OR 0.39, 95% CI 0.17–0.93, *p* = 0.035). Postnatal steroid exposure was associated with pulmonary hemorrhage (OR 4.59, 95% CI 1.01–20.78, *p* = 0.048). However, this finding likely reflects underlying disease severity, survivor bias, and confounding by indication rather than a direct causal effect. PDA diameter was not independently associated with pulmonary hemorrhage after multivariable adjustment (OR 3.14, 95% CI 0.82–12.07, *p* = 0.09). Affected neonates frequently exhibited progressive clinical deterioration characterized by increasing ventilatory support requirements, inotropic support, and fluid imbalance during the 24 h preceding pulmonary hemorrhage. Rescue surfactant administration during or shortly after the hemorrhagic episode was associated with mortality among affected neonates (OR 11.20, 95% CI 1.55–80.9, *p* = 0.017), although this finding should be interpreted cautiously because it may reflect underlying disease severity. Findings were consistent in sensitivity analyses.

**Conclusions:**

Pulmonary hemorrhage was associated with a recognizable pattern of progressive cardiorespiratory deterioration rather than isolated static clinical characteristics. Increasing respiratory support requirements, hemodynamic instability, and signs of fluid imbalance were common clinical features preceding the hemorrhagic event. These findings highlight a potential period of evolving clinical instability before pulmonary hemorrhage and provide a foundation for future prospective studies aimed at improving risk recognition and clinical monitoring in vulnerable neonates.

**Supplementary Information:**

The online version contains supplementary material available at 10.1186/s12887-026-07190-x.

## Key messages

### What is already known on this topic?

- Pulmonary hemorrhage is a devastating neonatal complication with persistently high mortality rates, particularly among extremely preterm infants.
- Existing literature has identified several perinatal and postnatal factors associated with pulmonary hemorrhage including prematurity, patent ductus arteriosus, and respiratory distress syndrome.
- However, contemporary data describing the clinical features observed immediately before pulmonary hemorrhage remain limited.

### What this study adds?

- This study identified a pattern of progressive clinical deterioration during the 24 hours preceding pulmonary hemorrhage, characterized by increasing ventilatory support requirements, inotropic support, and signs of fluid imbalance.
- Clinical features suggestive of fluid overload were frequently observed before pulmonary hemorrhage, supporting the need for prospective studies incorporating objective assessments of fluid balance.
- These findings provide hypothesis-generating observations regarding the evolution of cardiorespiratory instability before pulmonary hemorrhage.

### How this study might affect research, practice, or policy?

- The findings highlight the importance of careful clinical monitoring in high-risk neonates and suggest that pulmonary hemorrhage may be preceded by a period of evolving cardiorespiratory instability.
- The observed associations between fluid imbalance, cardiorespiratory deterioration, and pulmonary hemorrhage warrant further prospective investigation.
- Rescue surfactant administration during the hemorrhagic episode was associated with mortality and may represent a marker of disease severity rather than a direct treatment effect.
- Future prospective studies are needed to validate these findings and clarify the temporal relationships between clinical deterioration, treatment exposures, and pulmonary hemorrhage.

## Introduction

Pulmonary hemorrhage is a rare but devastating complication in neonates. It is characterized by acute pulmonary bleeding that leads to rapid respiratory deterioration and high mortality particularly among preterm and extremely low birth weight infants [1–3]. Despite advances in neonatal intensive care, outcome remains poor with reported incidence ranging from 1 to 12 per 1,000 live births [1–3]. The underlying pathophysiology is thought to involve a complex interplay between structurally immature pulmonary vasculature, hemodynamic instability, and inflammatory processes. A wide range of perinatal and postnatal factors have been implicated in the development of neonatal pulmonary hemorrhage including prematurity, patent ductus arteriosus (PDA), respiratory distress syndrome (RDS), surfactant therapy, and coagulation disorders [2, 3]. In addition, perinatal conditions such as chorioamnionitis, sepsis, intrauterine growth restriction may contribute to its occurrence [1–3].

However, important knowledge gaps remain. First, the relationship between fluid balance, acute hemodynamic fluctuations, and pulmonary hemorrhage has not been systematically evaluated in contemporary neonatal intensive care settings. In particular, little is known about the clinical trajectory preceding pulmonary hemorrhage and whether signs consistent with fluid overload represent antecedent physiological disturbances or early manifestations of the hemorrhagic process itself. Second, while PDA has long been associated with pulmonary hemorrhage, the relative contribution of ductal hemodynamic significance, ductal size and treatment exposure remains unclear in current literature. Third, much of the available evidence is derived from older cohorts or studies with limited sample sizes restricting its applicability to modern neonatal practice.

In this context, the present study aimed to evaluate a 10-year single-center cohort of neonates with pulmonary hemorrhage, with a particular focus on characterizing perinatal and postnatal clinical features, identifying factors associated with pulmonary hemorrhage, and describing the clinical trajectory preceding the hemorrhagic event. Uniquely, this study incorporates a detailed analysis of clinical changes within the 24 h preceding pulmonary hemorrhage, providing insight into the evolving clinical course preceding pulmonary hemorrhage. In addition, we performed a comprehensive evaluation of PDA-related parameters including ductal diameter and treatment exposure to better understand their relationship with pulmonary hemorrhage. Furthermore, contemporary therapeutic approaches including recombinant activated factor VII (rFVIIa) and tranexamic acid were examined, offering practical insights into current management strategies.

## Methods

### Study design and patient selection

This retrospective frequency-matched case–control study was conducted in a tertiary-level neonatal intensive care unit (NICU) following approval from the Institutional Ethics Committee. Neonates diagnosed with pulmonary hemorrhage between 2013 and 2023 constituted the case group. For each pulmonary hemorrhage case, one control neonate without pulmonary hemorrhage was selected from infants hospitalized in the same NICU during the same calendar month. Matching was performed based on birth weight (± 200 g) and gestational age (± 1 week). When more than one eligible control was available, the infant with the closest gestational age and birth weight was selected without consideration of clinical course.

Inclusion criteria comprised all neonates who met the diagnostic criteria for pulmonary hemorrhage and had a matched control. Exclusion criteria were survival for less than 12 h, chromosomal anomalies, transfer to another hospital, or incomplete clinical records.

### Study objectives and data collection

The primary objectives of this study were to characterize the clinical features and prognosis of neonates with pulmonary hemorrhage, describe the clinical features observed within the 24 h preceding hemorrhage onset, evaluate PDA-related clinical characteristics, and assess management strategies. Clinical variables recorded during the 24 h preceding pulmonary hemorrhage were evaluated only among affected neonates to characterize the clinical course before the event. Because a comparable index time point could not be assigned to control infants and the timing of certain postnatal exposures could not always be established, these analyses were considered descriptive and interpreted as associations rather than confirmed antecedent risk factors.

Clinical data were retrieved from electronic medical records and patient charts. Perinatal and antenatal factors including premature rupture of membranes and/or chorioamnionitis, oligohydramnios, intrauterine growth restriction, and maternal comorbidities were recorded. Postnatal clinical variables included RDS, ventilatory support, hemodynamic status, intraventricular hemorrhage (IVH), bronchopulmonary dysplasia (BPD), sepsis, in-hospital mortality, hemodynamically significant PDA (HsPDA), and treatment strategies was performed. Clinical and laboratory findings such as platelet counts, coagulation parameters, and liver and renal function tests were also collected. Therapeutic interventions included transfusions, ventilator setting adjustments, high-frequency ventilation, surfactant administration, endotracheal adrenaline, and inotropic support. In addition to conventional supportive measures, rescue therapies including rFVIIa and tranexamic acid were recorded. These agents were administered in selected cases unresponsive to standard treatments, including blood transfusion, ventilatory management, vitamin K administration, and hemodynamic support. rFVIIa was introduced in 2022, followed by tranexamic acid in 2023. Therefore, their use was limited to the later years of the study period. Furthermore, subgroup analyses were conducted to compare early- and late-onset pulmonary hemorrhage and to evaluate factors associated with mortality.

### Definitions

Definitions were based on established and widely accepted criteria to ensure data consistency throughout the 10-year study period and to allow direct comparability with previous studies in the literature.

Massive pulmonary hemorrhage was defined by the presence of bloody endotracheal or upper airway secretions accompanied by acute clinical deterioration and supportive radiologic findings when available [2]. Acute clinical deterioration was operationally defined as the need for intubation, a ≥ 20% increase in FiO₂, a ≥ 1 cmH₂O increase in inspiratory pressure, or an acute hematocrit drop exceeding 10%. Pulmonary hemorrhage occurring within the first 7 days of life was defined as early-onset pulmonary hemorrhage [1–3]. Management of pulmonary hemorrhage was based on unit protocols and included optimization of ventilatory support, transfusion of blood products when clinically indicated, correction of coagulopathy, and hemodynamic stabilization. High-frequency oscillatory ventilation, endotracheal adrenaline, and rescue surfactant therapy administered during the hemorrhagic episode or shortly thereafter were considered in selected patients with severe respiratory compromise, while rFVIIa and tranexamic acid were reserved for refractory cases unresponsive to conventional management.

The severity of IVH was classified according to the Volpe classification, necrotizing enterocolitis was staged using the Modified Bell criteria, and sepsis was evaluated according to the EMA neonatal sepsis scoring system [4–6].

BPD was defined as the need for supplemental oxygen for at least 28 days after birth [7]. Postnatal corticosteroids were administered according to the DART protocol to neonates who remained intubated on mechanical ventilation for more than one week and in whom failure to extubate was not attributable to conditions such as patent ductus arteriosus or sepsis [8].

HsPDA was diagnosed based on the overall echocardiographic assessment of ductal hemodynamic significance including ductal size, shunt characteristics, and evidence of increased pulmonary blood flow without requiring the presence of all individual criteria in every case. Echocardiographic evaluations were performed as part of routine clinical care typically within the first 12–72 h of life depending on gestational age and clinical status, and were subsequently repeated when clinically indicated.

Hypervolemia was defined as excessive weight gain and/or positive fluid balance accompanied by clinical signs of volume overload, including worsening respiratory status, increased oxygen requirement, generalized edema, or radiographic pulmonary congestion. Hematocrit decline was excluded to avoid circularity with the diagnosis of pulmonary hemorrhage. Because no universally accepted neonatal definition of hypervolemia exists, a pragmatic clinical definition based on routinely documented indicators of fluid overload was used.

Liver dysfunction and renal dysfunction were defined according to routine biochemical and clinical indicators of impaired organ function.

### Statistical analyses

All statistical analyses were performed using IBM SPSS Statistics software (Version 25.0; IBM Corp., Armonk, NY, USA). As most continuous variables were not normally distributed, they were expressed as median (25th–75th percentile) and compared using the Mann–Whitney U test. Categorical variables were presented as counts and percentages and compared using the chi-square test or Fisher’s exact test, as appropriate.

Because individual matched pairs were not retained as analytical strata, conventional logistic regression models were used instead of conditional logistic regression. Variables with a p-value < 0.10 in univariate analyses and those considered clinically relevant were included in the multivariate analysis. Potential multicollinearity among candidate predictors was assessed using variance inflation factor (VIF) and tolerance statistics before multivariable modeling. No significant multicollinearity was identified (all VIF values < 1.3 and all tolerance values > 0.80). Multivariate logistic regression analyses were performed to identify variables independently associated with pulmonary hemorrhage and mortality. Results were reported as odds ratios (ORs) with 95% confidence intervals (CIs). Study period was considered as a potential confounder to account for temporal changes in NICU practices. To evaluate the robustness of the findings, a sensitivity analysis was performed by restricting the cohort to the most recent period (2021–2023). The consistency of effect direction and magnitude between the primary and sensitivity analyses was used to evaluate the stability of the results. A two-sided p-value < 0.05 was considered statistically significant.

Missing data were confined to BPD and postnatal steroid exposure. BPD could be evaluated only among infants who survived beyond 28 days of life, whereas postnatal steroid exposure was assessed only in infants meeting predefined clinical eligibility criteria. Accordingly, analyses involving these variables were restricted to evaluable patients (complete-case analysis), and no imputation procedures were performed.

### Patient and public involvement

Patients and/or the public were not involved in the design, conduct, reporting, or dissemination plans of this research.

## Results

### Study cohorts

A total of 5610 preterm and term neonates were admitted to our NICU between 2013 and 2023. Of these, 1135 were born before 32 weeks’ gestation. A total of 72 neonates were diagnosed with pulmonary hemorrhage. After applying exclusion criteria [survival < 12 h (*n* = 3), chromosomal anomalies (*n* = 3), transfers to another hospital (*n* = 2), incomplete medical records (*n* = 4)], 60 pulmonary hemorrhage cases remained. Each case was matched to a control neonate admitted within the same month. Accordingly, among higher-risk infants born before 32 weeks, the incidence of pulmonary hemorrhage was 4.1%.

### Baseline and postnatal characteristics

Baseline and postnatal characteristics of the study population are presented in Table 1. Gestational age and birth weight were comparable between groups by design, as these variables were used for control selection. Antenatal corticosteroid exposure was significantly lower in the pulmonary hemorrhage group than in controls (65% vs. 85%, *p* = 0.02), whereas no other antenatal or intrapartum variables were significantly associated with pulmonary hemorrhage (Table 1).

**Table 1:** Baseline and early postnatal characteristics of the study population

	Pulmonary hemorrhage (*n* = 60)	Control (*n* = 60)	*p*
Antenatal and perinatal characteristics			
**Gestational age (weeks)**,** median (25P–75P)**	28 (27–31)	29 (26–31)	0.98
**Birth weight (g)**,** median (25P–75P)**	942 (722–1692)	1095 (806–1317)	0.97
**5-minute APGAR**,** median (25P–75P)**	7 (5–8)	7 (6–8)	0.09
**Small for gestational age**,** n (%)**	33 (55)	41 (68)	0.13
**Antenatal corticosteroid use**,** n (%)**	39 (65.0)	51 (85.0)	0.02
**Preeclampsia/Eclampsia**,** n (%)**	18 (30.0)	28 (46.7)	0.09
**PROM > 18 h and/or chorioamnionitis**,** n (%)**	24 (40.0)	23 (38.3)	0.99
**Oligohydramnios/Anhydramnios**,** n (%)**	16 (26.7)	20 (33.3)	0.24
**Perinatal aspyhixia**,** n (%)**	9(15)	3(5)	0.068
**Delivery room advanced resuscitation**, n (%)*	4(6.7)	1(1.7)	0.36
**Maternal infection**,** n (%)**	16(26.7)	8(13.3)	0.068
**Placental disorders**,** n (%)**	12(20)	8(13.3)	0.32
**Postnatal clinical characteristics**			
**Surfactant treatment for respiratory distress syndrome**,** n (%)**	55 (91.7)	44 (73.3)	0.01
**Severe IVH (≥ grade 2)**,** n (%)**	14 (23.3)	6 (10.0)	0.04
**Necrotizing enterocolitis (≥ stage 2)**,** n (%)**	9 (15.0)	8 (13.0)	1.00
**BPD**,** n (%)**^†^	12 (54.5)	13 (25.0)	0.02
**Postnatal steroid use for BPD prophylaxis**,** n (%)**^†^	17 (73.9)	16 (30.8)	0.001
**Culture-proven sepsis**,** n (%)**	14 (23.3)	8 (13.3)	0.15
**All-cause mortality**,** n (%)**	42 (70)	10 (16.7)	< 0.001

† Accordingly, analyses involving BPD were restricted to evaluable patients (*n* = 74; pulmonary hemorrhage: *n* = 22, control: *n* = 52). Postnatal steroid exposure was assessed only among infants meeting predefined clinical eligibility criteria and was therefore analyzed in evaluable patients (*n* = 75; pulmonary hemorrhage: *n* = 23, control: *n* = 52). Percentages were calculated based on available data for each variable. *PROM* premature rupture of membranes, *IVH* intraventricular hemorrhage, *BPD* bronchopulmonary dysplasia. *Chest compression and epinephrine

Among postnatal variables, surfactant administration for RDS (91.7% vs. 73.3%, *p* = 0.01), severe IVH (23.3% vs. 10.0%, *p* = 0.04), BPD (54.5% vs. 25.0%, *p* = 0.02), and postnatal steroid use for BPD prophylaxis (73.9% vs. 30.8%, *p* = 0.001) were more frequent among neonates with pulmonary hemorrhage than in controls. While the overall frequency of hsPDA did not differ significantly between groups (51.7% vs. 36.7%, *p* = 0.12), PDA diameter was significantly greater in neonates with pulmonary hemorrhage (1.8 [1.4–2.5] vs. 1.4 [1.1–2.0] mm, *p* = 0.01). Among treated infants, neither the number of PDA treatment courses nor the distribution of treatment modalities differed significantly between groups (Table 2). Other antenatal and postnatal variables were similar between the groups (Table 1).

**Table 2:** Patent ductus arteriosus (PDA)–related characteristics in neonates with and without pulmonary hemorrhage

	Pulmonary hemorrhage (*n* = 60)	Control (*n* = 60)	*p*
Hemodynamically significant PDA, *n* (%)	31 (51.7)	22 (36.7)	0.12
**PDA diameter (mm)**,** median (25P–75P)**	1.8 (1.4–2.5)	1.4 (1.1–2.0)	0.016
**PDA ligation**,** n (%)**	3 (5.0)	1 (1.7)	0.61
**Among infants receiving PDA-directed medical treatment**			
**PDA treatment course (n)**,** median (25P–75P)**	1 (1–1)	1 (1–2)	0.92
**Paracetamol monotherapy**,** n (%)**	17 (54.8)	14 (63.6)	0.58
**Ibuprofen monotherapy**,** n (%)**	8 (25.8)	2 (9.1)	0.16
**Combined therapy (paracetamol + ibuprofen)**,** n (%)**	6 (19.4)	6 (27.3)	1.00

Multivariable logistic regression analysis demonstrated an independent association between antenatal corticosteroid exposure and a lower likelihood of pulmonary hemorrhage (OR 0.399, 95% CI 0.170–0.935, *p* = 0.035). Postnatal steroid exposure remained associated with pulmonary hemorrhage in the adjusted model (OR 4.591, 95% CI 1.014–20.784, *p* = 0.048) (Table 3).

**Table 3:** Multivariate logistic regression analysis of variables associated with pulmonary hemorrhage

Variable	OR	95% CI	*p*
**Antenatal corticosteroid use**	0.399	0.170–0.935	0.03
**Postnatal steroid use for BPD prophylaxis**	4.591	1.014–20.784	0.04
**Patent ductus arteriosus diameter (mm)**	3.144	0.819–12.072	0.09

*BPD* bronchopulmonary dysplasia

### Clinical trajectory during the 24 h preceding pulmonary hemorrhage

**Fig. 1:**
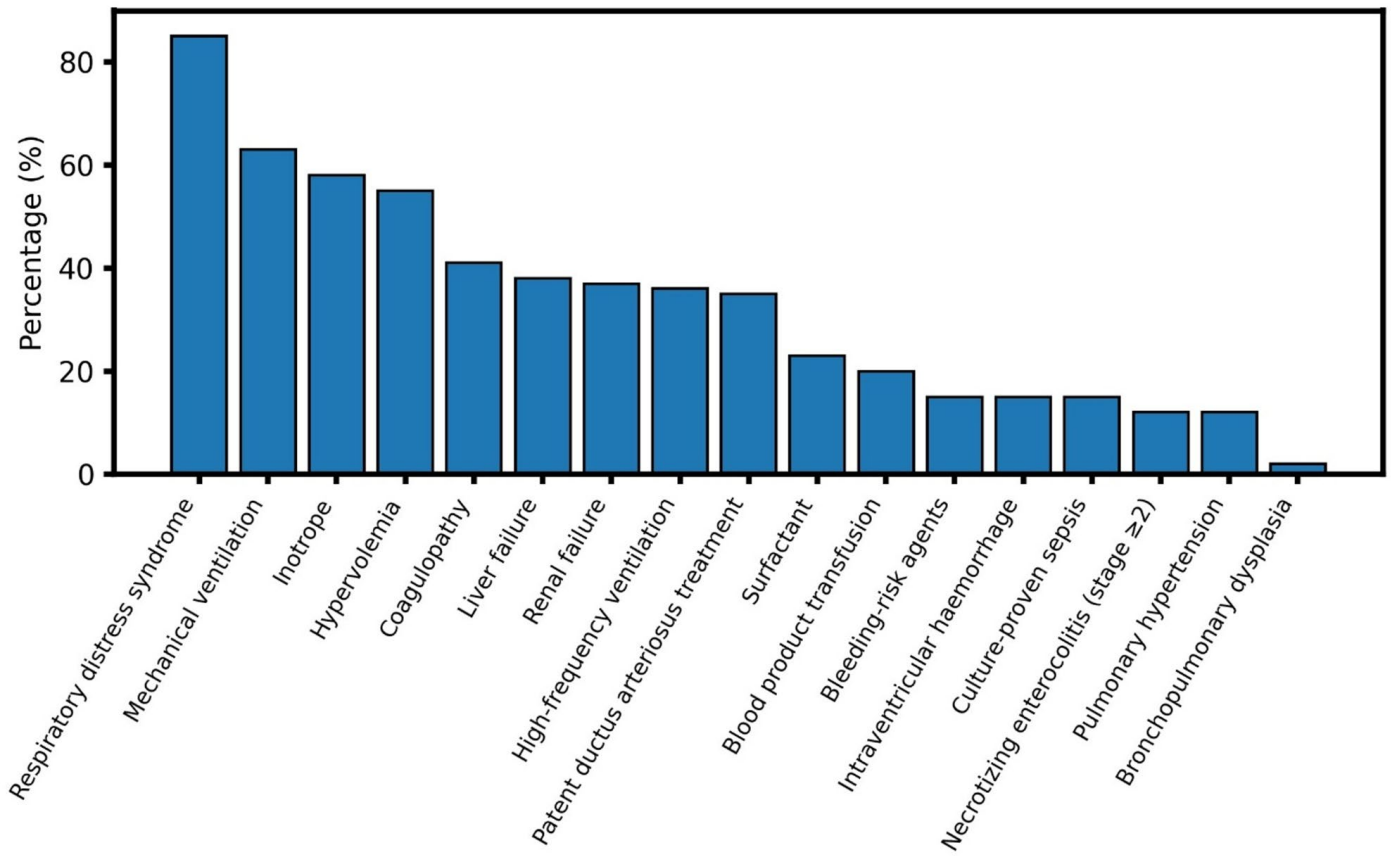
Clinical findings and interventions observed during the 24 h preceding pulmonary hemorrhage

During the 24 h preceding pulmonary hemorrhage, many neonates exhibited progressive clinical deterioration. Hypervolemia was present in 55% of cases, 63% required mechanical ventilation, and 58% received inotropic support (Fig. 1).

### Timing of pulmonary hemorrhage

Among the 60 neonates who experienced massive pulmonary hemorrhage, 43 (71.7%) had onset of hemorrhage within the first seven days of life (early neonatal period), while 17 (28.3%) developed hemorrhage after the first week (late onset). There were no significant differences between early- and late-onset pulmonary hemorrhage in terms of gestational age [28 (26–33) vs. 29 (27–30.5) weeks, *p* = 0.55] or birth weight [910 (700–1750) vs. 960 (810–1689.5) g, *p* = 0.29]. Although pulmonary hemorrhage–related mortality did not differ significantly between early- and late-onset groups (58.1% vs. 41.2%, *p* = 0.368), overall mortality was significantly higher in early-onset pulmonary hemorrhage (81.4% vs. 41.2%, *p* = 0.006).

### Mortality data

Overall mortality was 70% (42/60), whereas pulmonary hemorrhage-related mortality was 53% (32/60). The median day of hemorrhage occurrence was day 3 (25P-75P: 2–7) among those who died and day 5 (25P-75P: 2–13) among survivors. Non-survivors had significantly lower gestational age (27 vs. 30 weeks; *p* = 0.03) and birth weight (792 vs. 1310 g; *p* = 0.02). Maternal infection (40.6% vs. 10.7%; *p* = 0.02) and hepatic dysfunction (53.1% vs. 21.4%; *p* = 0.02) were also more prevalent among non-survivors. Additionally, use of inotropes (75% vs. 39.3%; *p* = 0.01) was more frequent in non-survivors (Table 4). In exploratory multivariable analysis, surfactant administration during the hemorrhagic episode was associated with mortality (OR 11.20, 95% CI 1.55–80.9, *p* = 0.017) (Table 5).

**Table 4:** Clinical characteristics of infants with pulmonary hemorrhage according to pulmonary hemorrhage-related mortality

	Pulmonary Hemorrhage-Related Survival		
	Survivors (n = 28)	**Non-survivors (n = 32)**	*P*
**Gestational age, median (25P-75P)**	30 (27-35.5)	27 (26–29)	0.03
**Birth weight (g), median (25P-75P)**	1310 (830–2310)	792(587–1257)	0.02
**Received antenatal corticosteroids, n (%)**	15 (53.6)	24(74)	0.08
**Maternal infection, n (%)**	3 (10.7)	13 (40.6)	0.02
**Hypervolemia, n (%)**	14 (50)	19 (59.4)	0.64
**Blood product transfusion, n (%)**	4 (14.3)	10 (31.3)	0.21
**Coagulopathy, n (%)**	11 (39.3)	14 (43.8)	0.93
**Inotrop use, n (%)**	11 (39.3)	24 (75)	0.01
**Respiratory distress syndrome, n (%)**	22 (78.6)	29 (90.6)	0.28
**Surfactant for pulmonary hemorrhage, n (%)**	12 (42.9)	20 (62.5)	0.12
**Necrotizing enterocolitis ≥ Stage 2, n (%)**	3 (10.7)	4 (12.5)	1.00
**Intraventricular hemorrhage, n (%)**	3 (10.7)	6 (18.8)	0.48
**Patent ductus arteriosus, n (%)**	15 (53)	16 (50)	0.78
**Culture-proven sepsis, n (%)**	10 (35.7)	2(6.1)	0.01
**Liver failure, n (%)**	6 (21.4)	17 (53.1)	0.02
**Renal failure, n (%)**	9 (32.1)	14 (43.8)	0.51

**Table 5:** Exploratory multivariate logistic regression analysis of factors identified within the 24 h prior to the pulmonary hemorrhage and associated with mortality

	Adjusted OR	95% CI	*p*
**Inotropic support**	1.58	0.13–19.0	0.71
**Surfactant for pulmonary hemorrhage**	11.20	1.55–80.9	0.01
**Liver failure**	9.46	0.52–170.2	0.12

### Therapeutic interventions for pulmonary hemorrhage

**Fig. 2:**
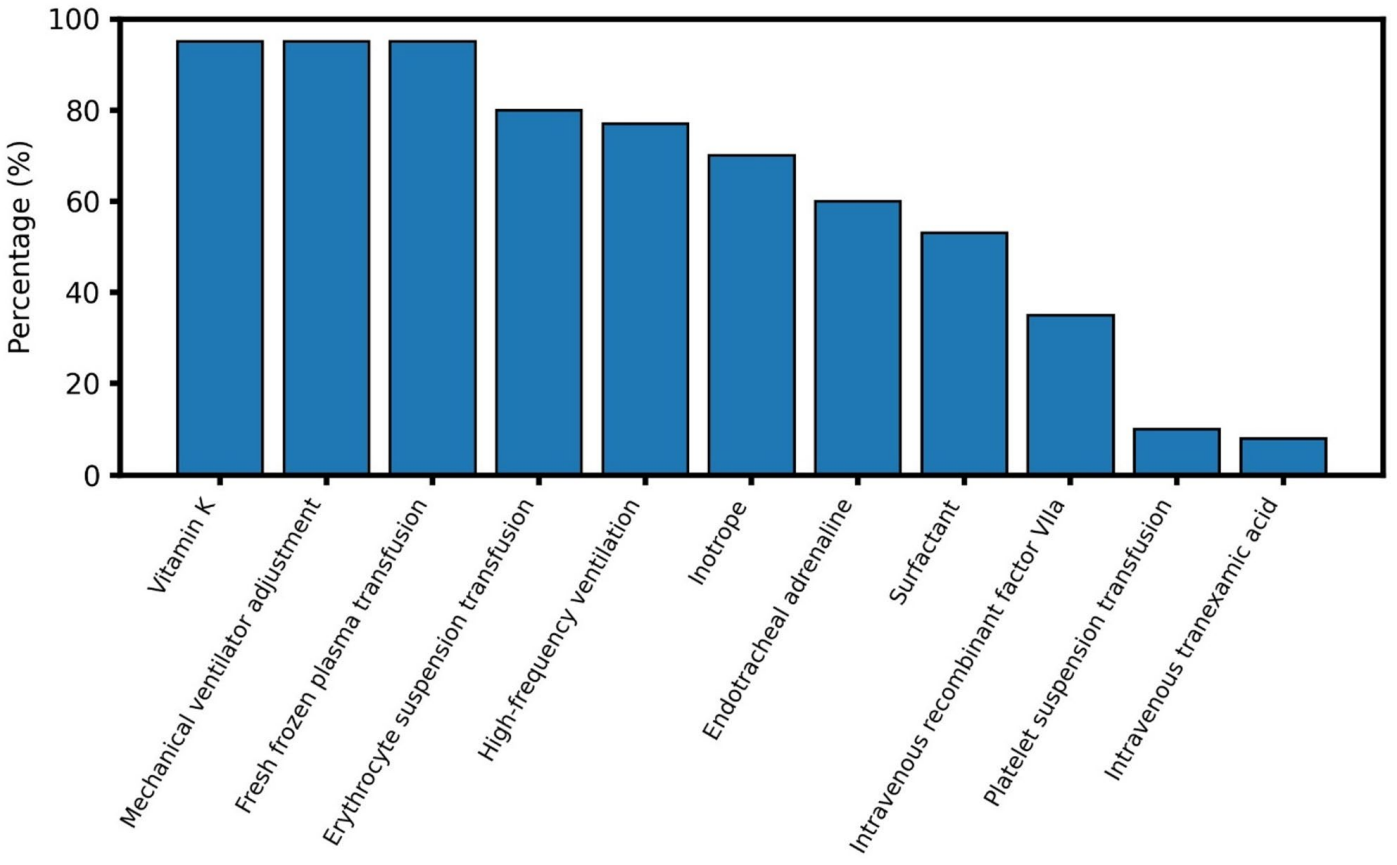
Interventions for massive pulmonary hemorrhage

Most infants with pulmonary hemorrhage required intensive interventions such as ventilator adjustment (95%), high-frequency ventilation (76%), endotracheal adrenaline (60%), and surfactant (53%). Fresh frozen plasma and vitamin K were administered to the majority. rFVIIa was used in 43% and tranexamic acid in 7% of refractory cases (Fig. 2).

## Discussion

We identified several clinically relevant factors associated with pulmonary hemorrhage in neonates in this 10-year single-center frequency-matched case–control study. The principal findings of our study can be summarized as follows: (i) antenatal corticosteroid exposure was associated with a lower occurrence of pulmonary hemorrhage; (ii) postnatal corticosteroid exposure was more frequently observed among neonates with pulmonary hemorrhage and remained associated with pulmonary hemorrhage in multivariable analyses, although this finding should be interpreted cautiously because of temporal ambiguity, survivor bias, and confounding by indication; (iii) PDA diameter was greater among infants with pulmonary hemorrhage, although this association did not remain statistically significant after multivariable adjustment; (iv) the 24 h preceding pulmonary hemorrhage were characterized by increasing ventilatory requirements, frequent inotropic support, and signs of fluid imbalance, suggesting evolving cardiorespiratory instability; and (v) rescue surfactant administration during the hemorrhagic episode was associated with mortality in exploratory analyses and is more likely to represent a marker of disease severity than a direct adverse effect.

Most bleeding episodes occurred within the first three postnatal days, consistent with previous reports [1, 2]. Pulmonary hemorrhage remains a life-threatening complication with persistently high mortality despite advances in neonatal care. In our cohort, pulmonary hemorrhage-related mortality was 53%, consistent with previously reported rates ranging from 50% to 68% [2, 9]. Furthermore, neonates with pulmonary hemorrhage experienced substantially higher overall mortality than matched controls (70% vs. 16.7%), a finding consistent with recent population-based data reported by Jani et al. [10]. The higher mortality observed among infants with early-onset pulmonary hemorrhage, despite similar hemorrhage-related mortality, suggests that early hemorrhage may reflect greater underlying illness severity rather than a direct determinant of death.

Antenatal corticosteroid exposure was associated with a lower likelihood of pulmonary hemorrhage, which is biologically plausible given its role in promoting lung maturation, improving alveolar stability, enhancing surfactant production, and reducing capillary fragility [2, 11]. In contrast, postnatal steroid exposure was more frequent among neonates with pulmonary hemorrhage and remained significant in multivariable analyses. However, this finding should be interpreted with considerable caution. Postnatal corticosteroids are typically administered to infants with more severe respiratory disease, prolonged ventilator dependence, and evolving bronchopulmonary dysplasia. Therefore, the observed association is likely influenced by confounding by indication, temporal ambiguity, and survivor bias. Rather than representing a direct causal risk factor, postnatal steroid exposure may serve as a marker of underlying disease severity in this high-risk population. Recent large-scale cohort studies including the Swedish study by Smedbäck et al., have highlighted the complexity of postnatal corticosteroid use and the need to balance potential respiratory benefits against systemic risks [12]. Accordingly, contemporary literature increasingly supports an individualized and physiology-informed approach to postnatal corticosteroid therapy rather than uniform treatment strategies [13].

Similarly, BPD should not be interpreted as a pre-event exposure because its diagnosis is established weeks after birth and can only be assessed in infants who survive long enough to meet diagnostic criteria. This interpretation is supported by recent evidence. In a cohort of very low birth weight infants, Pan et al. reported that although BPD was more common among infants with pulmonary hemorrhage in unadjusted analyses, pulmonary hemorrhage was not independently associated with either BPD or moderate-to-severe BPD after adjustment for confounding factors [14]. These findings suggest that the observed relationship between pulmonary hemorrhage and BPD may primarily reflect shared underlying disease severity. These results should therefore be interpreted within the broader context of evolving respiratory disease in extremely vulnerable neonates.

The present study provides a nuanced perspective on the relationship between hsPDA and pulmonary hemorrhage. Although PDA diameter was greater among infants with pulmonary hemorrhage in univariate analyses, neither the frequency of hsPDA nor PDA treatment characteristics differed significantly between groups. Furthermore, the association between PDA diameter and pulmonary hemorrhage did not remain statistically significant after multivariable adjustment (OR 3.14, 95% CI 0.82–12.07, *p* = 0.09). Therefore, the observed association between PDA diameter and pulmonary hemorrhage should be considered exploratory and interpreted with caution. Previous studies have reported associations between PDA and pulmonary hemorrhage in preterm infants, suggesting that increased pulmonary blood flow may contribute to pulmonary vascular vulnerability [14, 15]. In addition, echocardiographic studies have demonstrated larger PDA diameters and evidence of increased pulmonary blood flow and systemic steal among infants who developed pulmonary hemorrhage, supporting a potential hemodynamic contribution to the condition [2]. However, our findings suggest that the independent contribution of PDA-related characteristics remains uncertain and may reflect broader interactions among pulmonary immaturity, respiratory disease severity, pulmonary overcirculation, and hemodynamic instability. Contemporary evidence also emphasizes the hemodynamic consequences of PDA rather than ductal patency or diameter alone when assessing clinical significance [16].

A key strength of this study is the evaluation of clinical changes within the 24 h preceding pulmonary hemorrhage. During this period, many neonates demonstrated progressive deterioration including increasing ventilatory requirements, hemodynamic instability requiring inotropic support, and signs of fluid imbalance. These findings support the concept that pulmonary hemorrhage is often preceded by a phase of evolving cardiopulmonary instability. These findings may reflect a phase of clinical deterioration preceding pulmonary hemorrhage. However, it is also possible that they represent manifestations of advanced systemic illness rather than early predictive markers. Therefore, causality cannot be inferred from these observations.

Hypervolemia was assessed using a composite clinical approach because standardized and consistently recorded quantitative fluid balance data were not available in this retrospective cohort. Although this approach may introduce subjectivity, similar methods have been used in neonatal studies where real-time fluid dynamics are difficult to quantify. Importantly, hypervolemia was not diagnosed on the basis of respiratory deterioration alone but required evidence suggestive of fluid accumulation such as excessive weight gain and/or positive fluid balance together with accompanying clinical findings. Nevertheless, we acknowledge that some components of the definition, particularly respiratory deterioration and radiographic findings, may overlap with early manifestations of pulmonary hemorrhage or general clinical deterioration. Therefore, protopathic bias and misclassification cannot be excluded, and hypervolemia should be interpreted as a frequently observed pre-hemorrhage clinical feature rather than definitive evidence of a causal risk factor. The absence of a universally accepted definition of hypervolemia in preterm neonates remains a recognized limitation in the literature. Future prospective studies incorporating objective fluid balance metrics such as cumulative fluid balance and weight-adjusted fluid intake are needed to validate these findings. Recent American Academy of Pediatrics reports have also highlighted the limited contemporary evidence regarding the interaction between fluid balance and PDA-related hemodynamics in modern NICU practice [17]. In this context, the frequent observation of signs suggestive of fluid overload before pulmonary hemorrhage generates the hypothesis that fluid balance and ductal hemodynamics may interact in the pathophysiology of this condition. However, this hypothesis requires prospective validation using objective measures of fluid status before any causal or potentially modifiable relationship can be inferred.

Transfusion exposure also emerged as an important clinical consideration. Approximately one-quarter of neonates received transfusion prior to pulmonary hemorrhage consistent with previous reports [9]. Potential mechanisms include volume overload, increased pulmonary blood flow, oxidative stress, and inflammation [18–20]. These observations warrant further investigation into the relationship between transfusion exposure, fluid balance, and pulmonary hemorrhage in high-risk neonates.

Management strategies in our cohort reflected the multifactorial nature of pulmonary hemorrhage and included ventilatory optimization, surfactant administration, vasoactive support, and hemostatic interventions. rFVIIa and tranexamic acid were used in selected refractory cases that did not respond to standard therapies. rFVIIa was introduced into clinical practice in our unit from 2022 onwards, followed by the use of tranexamic acid from 2023. Our findings are consistent with our previous case series describing the off-label use of rFVIIa in neonates with pulmonary hemorrhage, in which this therapy was primarily reserved for severe, refractory cases [21]. However, evidence regarding the safety and efficacy of these agents in neonatal pulmonary hemorrhage remains limited, and their use should be considered on a case-by-case basis. Importantly, the introduction of these therapies during the later years of the study period may reflect evolving clinical practice, and although sensitivity analyses demonstrated consistent findings, potential temporal effects cannot be entirely excluded.

An additional clinically relevant observation in our study was the frequent use of fresh frozen plasma during the management of pulmonary hemorrhage. This likely reflects the urgent nature of the condition, where rapid clinical deterioration necessitates immediate intervention, often before standard laboratory coagulation results become available. We speculate that delays in obtaining coagulation parameters may have contributed to a more liberal use of empirical transfusion strategies in this setting. In response to these findings, point-of-care coagulation testing was subsequently implemented in our unit to facilitate more timely and targeted management. Although this change was not formally evaluated within the scope of the present study, it highlights how observational data may inform improvements in clinical practice. Future studies are needed to assess whether such strategies can optimize transfusion practices and improve outcomes in neonates with pulmonary hemorrhage.

Surfactant administration during the hemorrhagic episode was associated with mortality in the multivariable model. Importantly, this variable referred to rescue surfactant administered during the pulmonary hemorrhage episode or within the first hours thereafter, rather than routine surfactant therapy for respiratory distress syndrome. However, given the lack of a significant univariate association, the wide confidence intervals, and the likelihood that surfactant was preferentially administered to infants with more severe respiratory compromise, this finding should be considered exploratory and interpreted as a potential marker of disease severity rather than evidence of a causal relationship.

Importantly, sensitivity analyses restricted to the most recent cohort confirmed the consistency of our findings, supporting their external validity despite potential temporal changes in NICU practices. A key strength of this study is the identification of dynamic clinical changes within the 24 h preceding pulmonary hemorrhage, highlighting a potentially critical window for early recognition of evolving clinical instability. In addition, the evaluation of hypervolemia within the context of contemporary NICU practice represents a relevant strength, addressing a recognized gap in the literature regarding fluid balance and its interaction with hemodynamic factors in modern NICU settings. Furthermore, the detailed evaluation of PDA-related parameters including ductal diameter and treatment exposure rather than a simple binary classification provides a more physiologically meaningful assessment of its role in pulmonary hemorrhage.

This study has several limitations. First, its retrospective single-center design precludes causal inference and does not eliminate the possibility of residual confounding. Second, some exposures, particularly postnatal corticosteroid therapy, are susceptible to confounding by indication and temporal ambiguity because they are preferentially administered to infants with more severe respiratory illness. In addition, variables such as BPD and postnatal corticosteroid exposure could only be evaluated in eligible surviving infants and may therefore be affected by survivor bias. Third, the 10-year study period may have encompassed changes in neonatal management including respiratory support strategies and PDA treatment practices. Finally, given the number of comparisons performed, the findings should be considered exploratory and hypothesis-generating and require confirmation in larger prospective studies.

## Conclusion

In conclusion, pulmonary hemorrhage in neonates was associated with a complex interplay of cardiorespiratory instability and intensive clinical interventions. Rather than isolated static clinical characteristics, affected neonates frequently exhibited progressive clinical deterioration during the 24 h preceding the hemorrhagic event. These observations suggest the presence of a period of evolving instability before pulmonary hemorrhage and support further investigation of its clinical significance. Given the retrospective design and the potential for temporal ambiguity, survivor bias, and residual confounding, these findings should be considered hypothesis-generating and require prospective validation before causal inferences can be made.

## Supplementary Information

Supplementary Material 1.

## Data Availability

The datasets generated and/or analyzed during the current study are not publicly available due to institutional data protection policies but are available from the corresponding author on reasonable request.

## Abbreviations

ALT: Alanine aminotransferase
AST: Aspartate aminotransferase
BPD: Bronchopulmonary Dysplasia
CI: Confidence Interval
EMA: European Medicines Agency
FiO₂: Fraction of inspired oxygen
IQR: Interquartile Range
IVH: Intraventricular Hemorrhage
MAP: Mean Airway Pressure
MV: Mechanical Ventilation
NICU: Neonatal Intensive Care Unit
OR: Odds Ratio
PDA: Patent Ductus Arteriosus
PH: Pulmonary Hemorrhage
POC: Point-of-care
RDS: Respiratory Distress Syndrome
rFVIIa: Recombinant activated factor VII
VIF: variance inflation factor
