# Supplementary material for: Clinical trajectory and determinants of pulmonary hemorrhage in neonates: a 10-year case–control study
Source: BMC Pediatr. 2026 Jul 18;26:715. doi: 10.1186/s12887-026-07190-x (PMC13445757; doi:10.1186/s12887-026-07190-x)
Supplement: Supplementary file 1 [file 12887_2026_7190_MOESM1_ESM.docx]

**Supplementary Table S1.** Sensitivity analysis restricted to neonates born between 2021 and 2023

|  | **OR** | **95% CI** | **p** |
| --- | --- | --- | --- |
| **Antenatal corticosteroid use** | 0.35 | 0.12 – 1.01 | 0.05 |
| **Postnatal steroid use for BPD prophylaxis** | 5.96 | 0.92 – 38.61 | 0.06 |
| **PDA diameter (mm)** | 1.70 | 0.34 – 8.42 | 0.51 |
